# 3D Quantitative Evaluation of Posture and Spine Proprioceptive Perception Through Instinctive Self-Correction Maneuver in Adolescent Idiopathic Scoliosis

**DOI:** 10.3389/fbioe.2021.663394

**Published:** 2021-06-01

**Authors:** Edyta Kinel, Moreno D’Amico, Piero Roncoletta

**Affiliations:** ^1^Chair of Rehabilitation and Physiotherapy, Department of Rehabilitation, University of Medical Sciences, Poznań, Poland; ^2^SMART LAB (Skeleton Movement Analysis and Advanced Rehabilitation Technologies), Bioengineering & Biomedicine Company Srl, San Giovanni Teatino, Italy; ^3^Department of Neuroscience, Imaging and Clinical Sciences, “G. D’Annunzio” University of Chieti-Pescara, Chieti, Italy

**Keywords:** scoliosis, proprioception, posture, spine, self-correction, stereophotogrammetry

## Abstract

**Background:**

Conservative treatment in the adolescent idiopathic scoliosis (AIS) population is based on individual proprioceptive and motor control training. Such training includes physiotherapeutic scoliosis-specific exercises (PSSEs) stimulating the individual capacity to perceive and control his/her posture, particularly the shape of the spine. However, limited knowledge about basic proprioception capability in AIS patients is reported in the literature.

**Questions:**

(1) How do AIS patients, who did not receive any previous specific postural education treatment, perceive their posture and 3D spine shape? Are they able to modify their posture and 3D spine shape correctly through an instinctive self-correction (ISCO) maneuver? (2) Are posture and ISCO maneuver ability gender dependent in AIS patients? (3) Do AIS patients present different posture and spine shape characteristics as well as different ISCO ability compared with the healthy young adult population?

**Methods:**

Cross-sectional observational study. 132 (75 females, 57 males) AIS patients’ posture and 3D spine shape have been measured comparing indifferent orthostasis (IO) (neutral erect posture) to ISCO using a non-ionizing 3D optoelectronic stereophotogrammetric approach. Thirteen quantitative biomechanical parameters described the AIS patients body posture. The statistical analysis was performed using a multivariate approach to compare genders in IO, ISCO, and AIS patients vs. healthy young adults–previously published data (57 females, 64 males).

**Results:**

Males (87.7%) and females (93.3%) of AIS patients were unable to modify posture and 3D spine shape globally. AIS patients gender differences were found in IO, ISCO, and the comparison vs. healthy young adults. When changes occurred, subjects could not focus and control their posture globally, but only in a few aspects at a time.

**Conclusion:**

Self-correction maneuver producing an improvement in body posture and spine shape is not instinctive and must be trained. In such characteristics, AIS patients are not so dissimilar to healthy young adults. Sagittal plane control is the highest, but ISCO in AIS patients led to worsening in this plane. Control at the lumbar level is neglected in both genders. Such outcomes support the necessity of customized PSSEs to treat AIS patients. The 3D stereo-photogrammetric approach is effective in quantitatively describing the subject’s posture, motor control, and proprioception.

## Introduction

The latest literature on conservative treatment in adolescent idiopathic scoliosis (AIS) patients is predominantly based on proper individual proprioceptive (patient’s awareness) and motor control training. Such training includes physiotherapeutic scoliosis-specific exercises (PSSEs) stimulating the individual capacity to perceive and control his/her posture, and particularly the shape of the spine. The aim is to reduce spine deformities, limitation of the functional spinal units, and prevent inappropriate posture, improving the stability of the spine through voluntary intervention (Monticone et al., 2014, 2016; Berdishevsky et al., 2016; Kuru et al., 2016; Negrini et al., 2018, 2019; Schreiber et al., 2019). The literature reports that relaxed postures, which are typically adopted, frequently exacerbate low back pain or deformities (O’Sullivan, 2000; O’Sullivan et al., 2002, 2006; Waongenngarm et al., 2015; Bańkosz and Barczyk-Pawelec, 2020; Jung et al., 2020). Usually, young people may be referred to rehabilitation services to enhance body posture consistency and raise awareness about proper posture value (Monticone et al., 2014; Negrini et al., 2018). Teaching the appropriate active self-correction is considered essential in the conservative treatment for idiopathic scoliosis (Czaprowski et al., 2014; Monticone et al., 2014, 2016; Negrini et al., 2018). It has been claimed that one of the factors evaluating the efficacy of corrective interventions for enhancing body posture is the ability to adopt and sustain a correctly balanced body posture when carrying out activities of daily living (Weiss et al., 2006; Monticone et al., 2014, 2016; Negrini et al., 2018). PSSEs are claimed to be more effective than “usual physiotherapy” (Monticone et al., 2014; Negrini et al., 2019) or standard-of-care (observation and bracing) (Kuru et al., 2016; Schreiber et al., 2019) in AIS care. For example, Monticone et al. (2014) used an individualized therapeutic plan involving active self-correction tailored to the type of curve scoliosis for AIS patients. The inclusion criteria for the selection of AIS patients were: Cobb angle of 10°–25°, a Risser sign of <2, and an age of >10 years. The intervention lasted until skeletal maturity had been reached (Risser sign 5). A control group with the same characteristics was provided with general exercises aimed at spinal mobilization, spinal deep muscles strengthening, and lower limb and back muscles stretching, as well as balancing (through proprioceptive training when standing) and walking exercises (mainly devoted to resistance and velocity training). Monticone et al., 2014 found that the individualized therapeutic plan of active self-correction and task-oriented exercises was superior to traditional exercises, leading to a significant improvement in reducing spinal deformities (decrease in Cobb angle of >5°) and enhancing the health-related quality of life (evaluated through the SRS-22 questionnaire) in patients with mild AIS. In contrast, control group subjects stayed stable or had worsening spinal deformities.

No significant changes in the health-related quality of life were documented in the control group. During follow-up, 1 year after the intervention ended, the PSSEs group remained stable, while there was a slight Cobb angle worsening in the control group. The same research group carried on a similar study analyzing adults with idiopathic scoliosis (main Cobb angle <35°) (Monticone et al., 2016). Even in this study, the individualized therapeutic plan involving active self-correction tailored to the type of curve scoliosis resulted superior to general physiotherapy in reducing the disability of adults with idiopathic scoliosis. Additionally, motor and cognitive rehabilitation led to improvement in dysfunctional thoughts, pain, and quality of life. As for the adolescents, changes were maintained for at least 1 year following the intervention. Though the evidence in such randomized controlled studies supports superior effectiveness of the PSSEs approach vs. traditional physiotherapy in AIS treatment, it is still a matter of open debate, which kind of approach should be preferred. Indeed, a recent review (Day et al., 2019) concluded that: there is insufficient evidence to suggest that PSSEs methods can effectively improve Cobb angles in patients with AIS compared with no intervention. On the other hand, a recent study in the healthy young adult population (D’Amico et al., 2018a) showed that: (1) instinctive posture proprioception and motor control do not produce significant global improvement of body posture and spine shape using an instinctive self-correction (ISCO) maneuver; (2) proper and effective self-correction maneuver has to be learned with specific postural training; (3) asymptomatic healthy young adults do not have an optimal posture (D’Amico et al., 2017b, 2018a). However, there is limited knowledge reported in the literature about basic proprioception capability in AIS patients. Therefore, it is essential to analyze whether the problematic management of upright posture in subjects with idiopathic scoliosis can be linked to a further reduction of proprioceptive abilities. From all the above, the following research questions are raised:

(1) How do AIS patients, who did not receive any previous specific postural education treatment, perceive their posture and 3D spine shape? Are they able to correct their posture and 3D spine shape through an ISCO maneuver? (ISCO maneuver was stimulated by asking the subject to assume the best correct self-perceived standing posture without adding any specific indication or feedback).
(2) Are posture and ISCO maneuver ability gender-related in AIS patients?
(3) Do AIS patients present with a different posture and spine shape characteristics and different ISCO ability compared with healthy young adults (D’Amico et al., 2018a)?

## Materials and Methods

### Design

The research presented here is a cross-sectional observational study. We have used a validated, innovative stereophotogrammetric method of 3D quantitative evaluation of the entire skeleton posture and spine shape utilizing an evidence-based medicine approach (D’Amico et al., 2017b, 2018a; Kinel et al., 2018).

The Ethics Committee of the University of Medical Sciences in Poznan, Poland, approved this study. Resolution number: 75/17. All parents of participants had signed a written informed consent before the data collection began.

Data collection took place between February 2017 and 2019.

### The Participants

Participants diagnosed with AIS were sent to undergo quantitative 3D posture evaluation by external qualified medical specialists in orthopedics and/or rehabilitation medicine. Before the measurement session, all the interviews and physical examinations were conducted by a single qualified physiotherapist with 16 years of experience (the first-named author) to ensure consistency.

The inclusion/exclusion criteria were as follows: diagnosis of AIS, Cobb angle ≥10° (Negrini et al., 2018); males and females 11–18 years old; no ongoing brace treatment; no neurologic problems; no history of any previous specific postural education treatment; no history of musculoskeletal system injury or surgery; body weight within the normal range [as classified by Centers for Disease Control and Prevention growth charts for children 2–20 years old (Growth Charts - Clinical Growth Charts, 2019)].

A cohort of 132 AIS patients (75 females and 57 males) was recruited at the Clinic of Rehabilitation, University of Medical Sciences, Poznan, Poland.

The performances of such AIS patients in ISCO are compared with those of 121 healthy young adults, 57 females and 64 males, selected in a previously published research (D’Amico et al., 2018a). AIS patients’ and healthy young adults’ characteristics are summarized in Table 1.

**TABLE 1:** Sample population characteristics: Total of 132 adolescent idiopathic scoliosis (AIS) patients and 121 healthy young adults.

	AIS patients				Healthy young adults			
Population Characteristics	Females (*n* = 75)		Males (*n* = 57)		Females (*n* = 57)		Males (*n* = 64)	
	Range	Mean (SD)	Range	Mean (SD)	Range	Mean (SD)	Range	Mean (SD)
Age (year)	11–18	14.1 (2.1)	11–18	14.2 (2.3)	19–34	23.5 ± 3.2	20–35	24.9 ± 3.9
Height (cm)	140–174	160.9 (6.9)	140–187	166.7 (11.5)	155–175	163.9 ± 5.3	164–190	178.3 ± 6.7
Weight (kg)	32–83	51.1 (9.2)	31–95	58.5 (13.5)	40–71	56.1 ± 7.0	50–90	71.8 ± 8.6
BMI (kg/m^2^)	14.8–28.7	19.7 (3.2)	14.5–32.8	20.8 (3.3)	15.6–24.8	20.8 ± 2.0	18.6–24.9	22.5 ± 1.6

### Instrumentation

**FIGURE 1:**
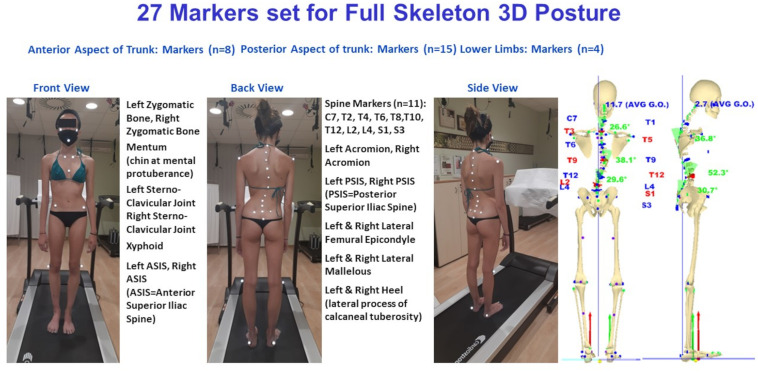
The 27 markers set used for 3D posture analysis. The front and back view body landmarks labeled by markers are listed. Full skeleton reconstruction is included. Underfoot loads are represented by vertical forces vectors (red vector on the left side, green vector on the right side).

Our experimental recordings were based on six TV cameras (resolution 1.3 Mpix, 120 fps, error range 0.3 mm, calibrated volume 3 × 3 × 2 meters GOALS^1^ (Global Opto-electronic Approach for Locomotion and Spine) stereophotogrammetric opto-electronic system derived from OptiTrack System^2^ (D’Amico et al., 2017a). We used one synchronous baropodometric platform^3^ to measure bilateral foot pressure maps and underfoot vertical forces exerted on each foot in a standing position. Data processing was performed using a software package named ASAP 3D Skeleton Model^1^. Such processing software implements a complete 3D parametric biomechanical human skeleton model (3D spine included). The bone anthropometric sizes of such a skeleton model fit the 3D opto-electronic measurements of a series of suitable body landmarks to assess the patient’s skeleton and posture. A 27 body landmarks protocol, labeled by passive retroreflective markers (Figure 1), has been set and tested extensively to analyze human posture in the clinical environment (D’Amico et al., 2017b, 2018a,b; Kinel et al., 2018).

### Acquisition Protocol

The standard trial session was aimed to define the participant’s indifferent orthostasis (IO) (i.e., maintaining the most natural erect posture). Afterward, the patient was asked to perform his/her instinctive self-corrected orthostasis (ISCO). The ISCO was stimulated by giving a generic command, i.e., requesting the patient to assume his/her best correct self-perceived standing posture without adding any specific indication or feedback. The same generic command was given in D’Amico et al. (2018a) for healthy young adults. As with healthy adults, AIS patients performed the ISCO maneuver effortlessly without reporting any kind of discomfort. Different positions of the feet can influence IO and ISCO postures. The subject was asked to align heels on a line parallel to the frontal plane and keep feet apart (without restricting feet directions) at about pelvis width (i.e., with feet under the hip joints projection) to avoid feet position influence. At least five subsequent 2-second lasting acquisitions at a 120 Hz sampling rate were recorded per each IO and ISCO condition. This way, a minimum of 1,200 3D measurements was averaged per each static postural stance. Averages were computed after defining a subject’s local coordinate system and the rotation needed in each acquired frame to align the subject’s skeleton 3D reconstruction within the absolute coordinate systems (D’Amico et al.,2017a,b, 2018a; Kinel et al., 2018).

**FIGURE 2:**
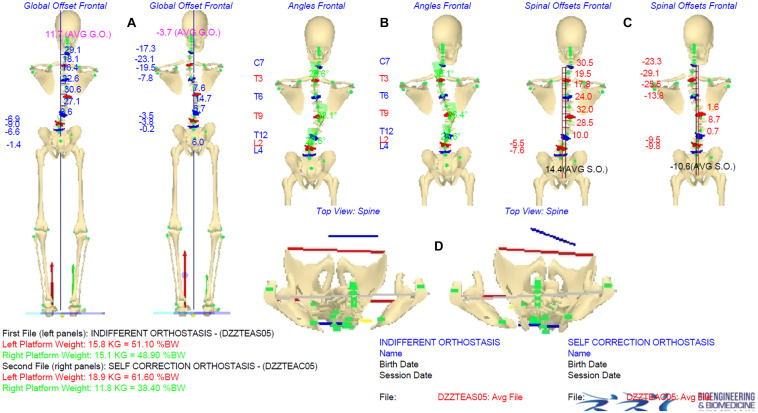
Indifferent orthostasis (IO) vs. instinctive self-correction (ISCO) maneuver comparison graphical report. Panels **(A–C)** show the comparison IO (left side) vs. ISCO (right side) in the frontal plane of averaged global offsets (AGO), spinal deformities, and Cobb angle values, and averaged spinal offsets (ASO), respectively. Panel **(D)** shows comparison IO (left side) vs. ISCO (right side) of rotations in the horizontal plane (shoulder girdle/pelvis). Underfoot loads are represented by vertical forces vectors (red vector on the left side, green vector on the right side) graphically showing the |ΔUL| parameter.

**FIGURE 3:**
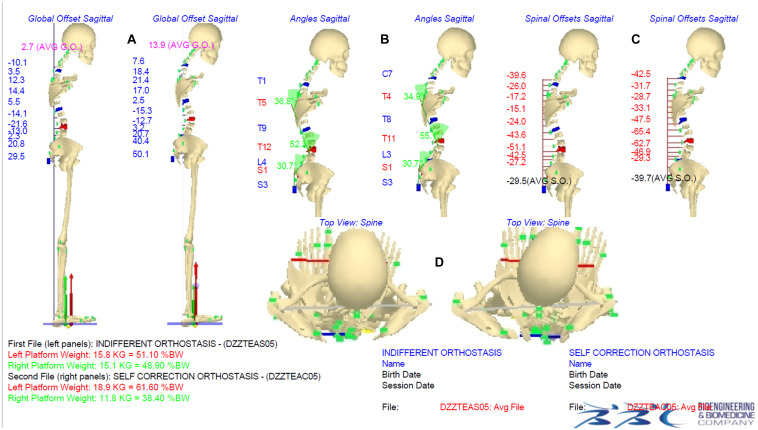
Indifferent orthostasis (IO) vs. instinctive self-correction (ISCO) maneuver comparison graphical report. Panels **(A–C)** show the comparison IO (left side) vs. ISCO (right side) in the sagittal plane of averaged global offsets (AGO-SG), thoracic kyphosis and lumbar lordosis angle values, and averaged spinal offsets (ASO-SG), respectively. Panel **(D)** shows comparison IO (left side) vs. ISCO (right side) of rotations in the horizontal plane (head, shoulder girdle/pelvis, feet positions). Underfoot loads are represented by vertical forces vectors (red vector on the left side, green vector on the right side) graphically showing the |ΔUL| parameter.

Figures 2, 3 show an example of a graphical report and the data elaboration outcomes of the IO vs. ISCO measurements comparison in the frontal and sagittal planes, respectively. A video showing the acquisition/elaboration processes can be found in the Supplementary Material (Supplementary Video 1).

### Outcome Measures

Based on the 3D biomechanical human skeleton model reconstruction, a set of 13 main significant parameters describing the three-dimensional nature of body posture was computed (D’Amico et al., 2017b, 2018a; Kinel et al., 2018). Such variables were subdivided into three groups, as reported in Table 2, where definitions and corresponding acronyms are given. It is worth noting that the signal processing procedure implemented to analyze the 3D spine shape automatically identifies all the curves present in the frontal and sagittal planes. In particular, based on measurements of the 11 labeled 3D spinous processes (from C7 down to S3 every second vertebra, Figure 1), data are interpolated using cubic splines in order to assess the position of each unlabeled spinous process and intervertebral disks. Smoothing is then performed on such noisy interpolated data. Next, the frontal and sagittal spine projections are derived from the filtered 3D analytical representation of the spine. Subsequently, frontal and sagittal spine shape curves are processed separately. The first and second derivative functions are assessed and used to identify the limit-vertebrae (i.e., vertebrae marking the beginning and the end of each identified curve) defined at curve inflection points (where the second derivative is equal to zero). From the values of the first derivative functions (i.e., the tangents’ value to the curve) at these inflection points, the Cobb and Kypho–Lordotic angle computations are straightforward per each determined curve. As it happens for the curve identified in the frontal plane, also the kyphosis and lordosis in the sagittal plane are appropriately identified according to the actual spine curvature spatial changes at the limit-vertebrae, i.e., they are no longer restricted to specific thoracic or lumbar anatomical regions (D’Amico et al., 1995, 2017b, 2018a; Kinel et al., 2018).

**TABLE 2:** List of considered parameters (definitions and corresponding acronyms) for indifferent orthostasis (IO) vs. instinctive self-correction (ISCO) comparison and summarizing indexes.

Global summarizing index	Parameters			Specific summarizing indexes
	Acronyms	Descriptions	Definitions	
GPI Global postural index	|ASO| (mm)	|Average frontal spinal offsets|	The ASO is the mean of the horizontal distances in the frontal plane of each labeled spine landmark respect to the vertical axis passing by S3; Absolute value of the average to disregard the side	FPI Frontal postural index
	|AGO| (mm)	|Average frontal global offsets|	The AGO is the mean of the horizontal distances in the frontal plane of each labeled spine landmark respect to the vertical axis passing through the middle point between heels; Absolute value of the average to disregard the side	
	|ΔASIS| (mm)	|ΔAnterior superior iliac spine|	Absolute ASIS height difference in frontal plane	
	|ΔPSIS| (mm)	|ΔPosterior superior iliac spine|	Absolute PSIS height difference in frontal plane	
	CA1; CA2 (degrees)	1°Cobb angle; 2°Cobb angle	Cobb angles of the two main “spinal deformities” found in the frontal plane	
	|PT (mm)|	|Pelvis torsion| = |(ΔASIS-ΔPSIS)|	Rotation of the right with respect to the left innominate bone. Rotations are intended around a horizontal axis running through the symphysis pubis. Absolute value to disregard the side	SPI Sagittal postural index
	ASO SG (mm)	Average sagittal spinal offsets	The ASO SG is the mean of horizontal distances in the sagittal plane of each labeled spine landmark respect to the vertical axis passing by S3; Negative value represent forward leaning	
	AGO SG (mm)	Average sagittal global offsets	The AGO SG is the mean of horizontal distances in the sagittal plane of each labeled spine landmark respect to the vertical axis passing through the middle point between heels; Negative value represent forward leaning	
	SA (degrees)	Sacral angle	The inclination of S1-S3 line with respect to the vertical line	
	TKA (degrees)	“Thoracic” Kyphosis angle	Kyphosis and lordosis are correctly identified following spine curvature spatial changes at inflection points, and so limit vertebrae are not strictly bounded to the specific anatomical region	
	LLA (degrees)	“Lumbar” Lordosis angle		
	|ΔUL| (%BW)	|ΔUnderfoot load|	Left vs. right sides body weight (BW) percentage difference. Absolute value to disregard the side	

We decided to consider the Cobb angle value of the two major curves (CA1, CA2, Table 2) for statistical analysis regarding the spinal deformities in the frontal plane.

### Group Statistical Analysis

Given the verified correlation (through correlation matrices computation) among the considered 13 quantitative postural parameters, the statistical analysis to compare females vs. males, IO vs. ISCO, and AIS patients vs. healthy young adults was performed using a multivariate approach. The paired samples Hotelling’s *T*^2^ test was applied in the IO vs. ISCO comparison. Conversely, for the females vs. males and AIS patients vs. healthy young adults, the independent samples Hotelling’s *T*^2^ test was used. After Hotelling’s tests were performed, the 95% confidence intervals were derived to assess the statistical significance of the difference of the means per each of the 13 quantitative parameters (Rencher, 2003). Such method is preferred over setting a battery of separate *t*-tests for each variable with Bonferroni correction on the type I error (α’ = α/k) because the latter approach does not take into account the correlation between the variables, and therefore, it results in an over-correction of the significance value α (Rencher, 2003). Female vs. male comparison was performed in IO to analyze eventual postural gender differences and subsequently in ISCO to investigate a possible different self-correction ability by gender. Comparisons were made between AIS patients and healthy young adults, both in IO and ISCO, to highlight any postural, proprioceptive, and motor control differences.

### Intra-Subject Statistical Analysis

At the intra-subject level, we investigated how the ISCO modified the subject’s posture by improving, worsening, or unchanging the original attitude. The comparison was performed through a *t*-test between the mean values of 13 considered quantitative parameters obtained per participant in the IO and the ISCO postures.

The actual postural parameter was classified “*Unchanged*” if there was no statistically significant difference.

Conversely, we defined the following as “*Improvement*”:

• *Frontal Plane parameters*: when the parameter values approached the optimal theoretical zero value during the ISCO (D’Amico et al., 2018a).
• *Sagittal Plane parameters*: in this case [except for pelvis torsion (|PT|) that should be zero], there are no theoretical optimal reference values, so we decided to consider the normative data determined in previous studies in healthy young adults, for IO and ISCO, as reference values to be approached (D’Amico et al., 2017b, 2018a; Kinel et al., 2018).
• |ΔUL| (*i.e., the difference of underfoot load between the feet*): the optimal theoretical condition is achieved when there is a perfect balance of underfoot load distribution between the left and right sides; therefore, there was “*Improvement*” when changes approached this condition.

“*Worsening*”: each time, during the self-correction (ISCO), a statistically significant change differed from the definitions of “*Improvement*,” it was concluded that a “*Worsening*” had occurred.

### Summarizing Indexes

A summarizing index was defined for each participant, assigning a +1, −1, or 0 scores when an “*Improvement*,” a “*Worsening*,” or “*Unchanged*” was respectively determined. Henceforth a “Global Postural Index” (GPI_i_) given by the sum of scores obtained for all variables for the ith participant was defined. The frontal plane index (FPI_i_) and the sagittal plane index (SPI_i_) were defined by the sum of the scores for the variables of the related group (D’Amico et al., 2017b, 2018a; Table 2).

Each of the summarizing indexes was regarded as “*Improvement*” if the summed parameters got a positive score ≥50% of the maximum obtainable positive score; conversely, “*Worsening*” if such sum got a negative score ≥50% of the maximum obtainable negative score; “*Unchanged*” in the other cases (D’Amico et al., 2017b, 2018a).

By counting the number of “*Improvement*,” “*Worsening*,” and “*Unchanged*” obtained for each participant in each parameter, it is possible to determine the percentages of “*Improvement*,” “*Worsening*,” and “*Unchanged*” achieved in the male and female subgroups.

### Power Analysis and Sample Size

Among all the tests, the most critical condition for power analysis is given by the independent Hotelling’s *T*^2^ test when applied to AIS patients vs. healthy young adults male groups comparison. In such a case, using GPower software (Faul et al., 2007), given the AIS patients (57 males and 75 females) and healthy young adults (64 males and 57 females) sample sizes, an effect size (Mahalanobis distance) *d* = 0.80 is granted (power = 80%, α = 5%, and *k* = 13 number of variates). Conversely, for the Hotelling’s *T*^2^ paired version (IO vs. ISCO in AIS patients), the effect sizes *d* = 0.62 for the male group and *d* = 0.53 for the female group are granted.

## Results

### Group Statistical Analysis

In the group statistical analysis, we investigated AIS patients gender differences both in IO and ISCO. Table 3 shows the results of the independent samples Hotelling *T*^2^ test between genders. By considering each variable’s confidence intervals, a statistically significant postural difference between genders both in IO and ISCO is determined. It is worth noting that the gender differences in the IO are in seven out of 13 (53.8%) of the considered postural parameters, while they are reduced to four (30.76%) in the ISCO since in such condition, the difference vanishes for the 2nd Cobb angle (CA2), the thoracic kyphosis angle (TKA), and the pelvic torsion (|PT|).

**TABLE 3:** AIS patients female vs. male comparisons in both IO and ISCO: Hotelling *T*^2^ tests results, 95% confidence intervals, and difference of means.

	Hotelling *T*^2^ test for independent samples: female vs. male in IO and ISCO comparison								
		IO (*n*1 = 57, *n*2 = 75, *k* = 13, *T*^2^ = 51.8, *p* = 8.2e-5, *d* = 1.26, power = 0.99)				ISCO (*n*1 = 57, *n*2 = 75, *k* = 13, *T*^2^ = 46.5, *p* = 3.1e–4, *d* = 1.19, power = 0.99)			
Parameter	Descriptions	Males Mean	Females Mean	Difference of Means	CI 95% lower ÷ upper	Males Mean	Females Mean	Difference of Means	CI 95% lower ÷ upper
|ASO| (mm)	|Average frontal spinal offsets|	8.1 ± 6.7	7.0 ± 4.9	1.07	−0.93 ÷ 3.07	7.3 ± 5.2	6.7 ± 4.7	0.60	−1.12 ÷ 2.31
|AGO| (mm)	|Average frontal global offsets|	12.4 ± 11.5	11.3 ± 7.4	1.14	−2.13 ÷ 4.41	10.8 ± 9.8	11.0 ± 7.8	−0.20	−3.23 ÷ 2.83
CA1 (degrees)	1°Cobb angle;	15.1 ± 6.9	16.4 ± 8.1	−1.28	−3.92 ÷ 1.35	14.3 ± 7.0	15.4 ± 7.6	−1.11	−3.66 ÷ 1.44
CA2 (degrees)	2°Cobb angle	10.0 ± 5.9	12.4 ± 7.1	−2.49*	−4.79 ÷−0.19	10.2 ± 5.8	11.6 ± 7.2	−1.38	−3.69 ÷ 0.94
TKA (degrees)	“Thoracic” Kyphosis angle	47.3 ± 8.5	43.3 ± 10.9	3.99*	0.54 ÷ 7.45	38.5 ± 10.4	35.2 ± 10.9	3.26	−0.47 ÷ 6.98
LLA (degrees)	“Lumbar” Lordosis angle	39.7 ± 8.3	43.1 ± 9.5	−3.36*	−6.49 ÷−0.23	40.1 ± 10.0	43.3 ± 10.2	−3.15*	−6.66 ÷−0.36
|ΔASIS| (mm)	|ΔAnterior superior iliac spine|	10.1 ± 8.2	7.8 ± 5.6	2.29	−0.10 ÷ 4.68	9.1 ± 7.1	7.7 ± 6.3	1.39	−0.92 ÷ 3.71
|ΔPSIS| (mm)	|ΔPosterior superior iliac spine|	6.5 ± 4.2	5.6 ± 4.5	0.91	−0.60 ÷ 2.43	6.1 ± 3.9	5.3 ± 3.8	0.76	−0.58 ÷ 2.11
|PT| (mm)	|Pelvis torsion| = | (ΔASIS-ΔPSIS)|	6.7 ± 5.6	5.0 ± 3.6	1.71*	0.13 ÷ 3.28	6.6 ± 5.1	5.5 ± 5.3	1.11	−0.69 ÷ 2.92
SA (degrees)	Sacral angle	16.8 ± 5.6	18.9 ± 6.3	−2.12*	−4.22 ÷−0.03	18.6 ± 5.0	21.1 ± 5.9	−2.49*	−4.41 ÷−0.56
ASO SG (mm)	Average sagittal spinal offsets	−14.1 ± 12.8	−24.2 ± 12.6	10.15*	5.75 ÷ 14.56	−21.7 ± 12.6	−30.7 ± 13.4	9.02*	4.48 ÷ 13.56
AGO SG (mm)	Average sagittal global offsets	−10.4 ± 23.3	−2.9 ± 19.0	−7.53*	−14.81 ÷−0.24	−14.9 ± 24.5	−6.7 ± 19.1	−8.10*	−15.61 ÷−0.59
|ΔUL| (%BW)	|ΔUnderfoot load|	6.3 ± 5.2	6.2 ± 3.8	0.09	−1.46 ÷ 1.64	6.9 ± 4.7	7.2 ± 4.2	−0.26	−1.81 ÷ 1.29

The * associated with bold numbers indicates the statistically significant differences of means.

Subsequently, we investigated the postural differences by gender in the IO vs. ISCO comparison through the Hotelling *T*^2^ test for paired samples (Table 4). The test demonstrated a statistically significant postural difference between indifferent vs. self-corrected orthostasis.

**TABLE 4:** Per-gender IO vs. ISCO comparisons in AIS patients: Hotelling *T*^2^ tests results, 95% confidence intervals, and difference of means.

	Hotelling *T*^2^ test for paired samples: per-gender IO vs. ISCO comparison								
		Males (*n* = 57, *k* = 13, *T*^2^ = 248.6, *p* = 4e-12, *d* = 2.08, power = 0.99)				Females (*n* = 75, *k* = 13, *T*^2^ = 123.0, *p* = 5.2e-9, *d* = 1.21, power = 0.99)			
Parameter	Descriptions	IO Mean	ISCO Mean	Difference of Means	CI 95% lower÷upper	IO Mean	ISCO Mean	Difference of Means	CI 95% lower ÷ upper
|ASO| (mm)	|Average frontal spinal offsets|	8.1 ± 6.7	7.3 ± 5.2	0.81	−0.41 ÷ 2.03	7.0 ± 4.9	6.7 ± 4.7	0.34	−0.56 ÷ 1.24
|AGO| (mm)	|Average frontal global offsets|	12.4 ± 11.5	10.8 ± 9.8	1.59	−1.09 ÷ 4.27	11.3 ± 7.4	11.0 ± 7.8	0.26	−1.10 ÷ 1.61
CA1 (degrees)	1°Cobb angle	15.1 ± 6.9	14.3 ± 7.0	0.84	−0.40 ÷ 2.08	16.4 ± 8.1	15.4 ± 7.6	1.01*	0.09 ÷ 1.94
CA2 (degrees)	2°Cobb angle	10.0 ± 5.9	10.2 ± 5.8	−0.27	−1.15 ÷ 0.60	12.4 ± 7.1	11.6 ± 7.2	0.84	−0.02 ÷ 1.70
TKA (degrees)	“Thoracic” Kyphosis angle	47.3 ± 8.5	38.5 ± 10.4	8.84*	6.42 ÷ 11.25	43.3 ± 10.9	35.2 ± 10.9	8.10*	5.20 ÷ 11.01
LLA (degrees)	“Lumbar” Lordosis angles	39.7 ± 8.3	40.1 ± 10.0	−0.41	−3.11 ÷ 2.29	43.1 ± 9.5	43.3 ± 10.2	−0.20	−2.07 ÷ 1.67
|ΔASIS| (mm)	|ΔAnterior superior iliac spine|	10.1 ± 8.2	9.1 ± 7.1	0.96	−0.28 ÷ 2.20	7.8 ± 5.6	7.7 ± 6.3	0.06	−1.06 ÷ 1.19
|ΔPSIS| (mm)	|ΔPosterior superior iliac spine|	6.5 ± 4.2	6.1 ± 3.9	0.38	−0.10 ÷ 0.87	5.6 ± 4.5	5.3 ± 3.8	0.23	−0.26 ÷ 0.73
|PT| (mm)	|Pelvis torsion| = |(ΔASIS-ΔPSIS)|	6.7 ± 5.6	6.6 ± 5.1	0.04	−0.87 ÷ 0.94	5.0 ± 3.6	5.5 ± 5.3	−0.56	−1.81 ÷ 0.69
SA (degrees)	Sacral angle	16.8 ± 5.6	18.6 ± 5.0	−1.82*	−3.13 ÷−0.52	18.9 ± 6.3	21.1 ± 5.9	−2.18*	−3.50 ÷−0.86
ASO SG (mm)	Average sagittal spinal offsets	−14.1 ± 12.8	−21.7 ± 12.6	7.60*	4.21 ÷ 10.99	−24.2 ± 12.6	−30.7 ± 13.4	6.47*	3.55 ÷ 9.38
AGO SG (mm)	Average sagittal global offsets	−10.4 ± 23.3	−14.9 ± 24.5	4.46	−0.36 ÷ 9.28	−2.9 ± 19.0	−6.7 ± 19.1	3.88*	0.67 ÷ 7.08
|ΔUL| (%BW)	|ΔUnderfoot load|	6.3 ± 5.2	6.9 ± 4.7	−0.64	−2.15 ÷ 0.87	6.2 ± 3.8	7.2 ± 4.2	−1.00	−2.42 ÷ 0.43

The * associated with bold numbers indicates the statistically significant differences of means.

As a final evaluation, AIS patients vs. healthy young adults were compared by gender (Table 5). In such a case, the Hotelling *T*^2^ test for independent samples demonstrated a statistically significant postural difference in all four comparisons.

**TABLE 5:** Per-gender AIS patients vs. healthy young adults comparisons in both IO and ISCO: Hotelling *T*^2^ tests results, 95% confidence intervals, and difference of means.

		Hotelling *T*^2^ test for independent samples: AIS patients vs. healthy young adults in IO and ISCO comparison							
		Females							
		IO (*n*1 = 75, *n*2 = 57, *k* = 13, *T*^2^ = 43.9, *p* = 6.0e-4, *d* = 1.16, power = 0.99)				ISCO (*n*1 = 75, *n*2 = 57, *k* = 13, *T*^2^ = 66.3, *p* = 2.1e-6, *d* = 1.43, power = 0.99)			
Parameter	Descriptions	AIS patients mean	Healthy young adults mean	Difference of Means	CI 95% lower÷upper	AIS patients mean	Healthy young adults mean	Difference of Means	CI 95% lower÷upper
|ASO| (mm)	|Average frontal spinal offsets|	7.0 ± 4.9	6.5 ± 4.6	0.46	−1.20 ÷ 2.12	6.7 ± 4.7	6.3 ± 4.1	0.35	−1.20 ÷ 1.89
|AGO| (mm)	|Average frontal global offsets|	11.3 ± 7.4	12.1 ± 8.1	−0.78	−3.46 ÷ 1.90	11.0 ± 7.8	11.0 ± 8.1	−0.01	−2.76 ÷ 2.75
CA1 (degrees)	1°Cobb angle;	16.4 ± 8.1	10.3 ± 5.0	6.09*	3.69 ÷ 8.49	15.4 ± 7.6	9.5 ± 4.8	5.92*	3.65 ÷ 8.19
CA2 (degrees)	2°Cobb angle	12.4 ± 7.1	7.5 ± 4.1	4.98*	2.89 ÷ 7.07	11.6 ± 7.2	7.2 ± 3.9	4.45*	2.36 ÷ 6.54
TKA (degrees)	“Thoracic” Kyphosis angle	43.3 ± 10.9	47.2 ± 8.6	−3.89*	−7.36 ÷−0.42	35.2 ± 10.9	40.8 ± 8.7	−5.63*	−9.12 ÷−2.15
LLA (degrees)	“Lumbar” Lordosis angle	43.1 ± 9.5	44.2 ± 9.7	−1.12	−4.45 ÷ 2.20	43.3 ± 10.2	43.7 ± 10.4	−0.44	−4.01 ÷ 3.13
|ΔASIS| (mm)	|ΔAnterior superior iliac spine|	7.8 ± 5.6	8.2 ± 5.5	−0.42	−2.37 ÷ 1.52	7.7 ± 6.3	8.0 ± 5.6	−0.32	−2.41 ÷ 1.77
|ΔPSIS| (mm)	|ΔPosterior superior iliac spine|	5.6 ± 4.5	4.8 ± 2.6	0.82	−0.50 ÷ 2.13	5.3 ± 3.8	4.7 ± 2.6	0.63	−0.54 ÷ 1.79
|PT| (mm)	|Pelvis torsion| = |(ΔASIS-ΔPSIS)|	5.0 ± 3.6	5.45 ± 3.9	−0.49	−1.78 ÷ 0.80	5.5 ± 5.3	5.6 ± 4.4	−0.04	−1.69 ÷ 1.61
SA (degrees)	Sacral angle	18.9 ± 6.3	17.3 ± 5.9	1.66	−0.48 ÷ 3.80	21.1 ± 5.9	18.2 ± 5.0	2.88*	0.95 ÷ 4.80
ASO SG (mm)	Average sagittal spinal offsets	−24.2 ± 12.6	−20.6 ± 11.9	−3.59	−7.87 ÷ 0.68	−30.7 ± 13.4	−23.5 ± 11.6	−7.18*	−11.58 ÷−2.78
AGO SG (mm)	Average sagittal global offsets	−2.9 ± 19.0	−1.8 ± 26.7	−1.09	−8.96 ÷ 6.79	−6.7 ± 19.1	−0.4 ± 26.9	−6.36	−14.29 ÷ 1.58
|ΔUL| (%BW)	|ΔUnderfoot load|	6.2 ± 3.8	5.1 ± 4.3	1.10	−0.29 ÷ 2.49	7.2 ± 4.2	5.4 ± 3.7	1.81*	0.41 ÷ 3.22

		**Males**							
		**IO (*n*1 = 57, *n*2 = 64, *k* = 13, *T*^2^ = 56.2, *p* = 3.7e-5, *d* = 1.36, power = 0.99)**				**ISCO (*n*1 = 57, *n*2 = 64, *k* = 13, *T*^2^ = 54.5, *p* = 5.7e-5, *d* = 1.34, power = 0.99)**			
**Parameter**	**Descriptions**	**AIS patients mean**	**Healthy young adults mean**	**Difference of Means**	**CI 95% lower÷upper**	**AIS patients mean**	**Healthy young adults mean**	**Difference of Means**	**CI 95% lower÷upper**

|ASO| (mm)	|Average frontal spinal offsets|	8.1 ± 6.7	6.2 ± 5.1	1.84	−0.29 ÷ 3.96	7.3 ± 5.2	5.8 ± 4.6	1.44	−0.32 ÷ 3.21
|AGO| (mm)	|Average frontal global offsets|	12.4 ± 11.5	11.6 ± 8.4	0.82	−2.78 ÷ 4.43	10.8 ± 9.8	12.8 ± 8.7	−1.92	−5.26 ÷ 1.41
CA1 (degrees)	1°Cobb angle	15.1 ± 6.9	11.5 ± 5.4	3.65*	1.45 ÷ 5.86	14.3 ± 7.0	10.4 ± 5.3	3.89*	1.67 ÷ 6.11
CA2 (degrees)	2°Cobb angle	10.0 ± 5.9	7.2 ± 4.3	2.72*	0.87 ÷ 4.56	10.2 ± 5.8	7.0 ± 4.7	3.23*	1.33 ÷ 5.12
TKA (degrees)	“Thoracic” Kyphosis angle	47.3 ± 8.5	45.1 ± 8.9	2.23	−0.92 ÷ 5.38	38.5 ± 10.4	36.4 ± 8.4	2.04	−1.35 ÷ 5.43
LLA (degrees)	“Lumbar” Lordosis angle	39.7 ± 8.3	32.8 ± 8.1	7.06*	4.09 ÷ 10.03	40.1 ± 10.0	32.3 ± 8.4	7.81*	4.49 ÷ 11.13
|ΔASIS| (mm)	|ΔAnterior superior iliac spine|	10.1 ± 8.2	7.5 ± 5.3	2.55*	0.09 ÷ 5.01	9.1 ± 7.1	7.6 ± 5.2	1.48	−0.74 ÷ 3.70
|ΔPSIS| (mm)	|ΔPosterior superior iliac spine|	6.5 ± 4.2	5.1 ± 2.2	1.42*	0.23 ÷ 2.60	6.1 ± 3.9	5.1 ± 2.2	1.01	−0.13 ÷ 2.14
|PT| (mm)	|Pelvis torsion| = |(ΔASIS-ΔPSIS)|	6.7 ± 5.6	5.3 ± 4.5	1.25	−0.57 ÷ 3.07	6.6 ± 5.1	5.6 ± 4.8	1.00	−0.78 ÷ 2.78
SA (degrees)	Sacral angle	16.8 ± 5.6	15.7 ± 5.5	1.11	−0.88 ÷ 3.10	18.6 ± 5.0	16.8 ± 5.5	1.83	−0.06 ÷ 3.72
ASO SG (mm)	Average sagittal spinal offsets	−14.1 ± 12.8	−14.0 ± 12.4	−0.06	−4.60 ÷ 4.48	−21.7 ± 12.6	−17.4 ± 13.5	−4.22	−8.93 ÷ 0.50
AGO SG (mm)	Average sagittal global offsets	−10.4 ± 23.3	−10.2 ± 21.5	−0.24	−8.29 ÷ 7.81	−14.9 ± 24.5	−8.8 ± 19.4	−6.09	−14.00 ÷ 1.83
|ΔUL| (%BW)	|ΔUnderfoot load|	6.3 ± 5.2	4.5 ± 3.8	1.74*	0.12 ÷ 3.37	6.9 ± 4.7	5.1 ± 4.5	1.84*	0.17 ÷ 3.50

The * associated with bold numbers indicates the statistically significant differences of means.

It is worth noting that only for a subset of parameters, the differences are present in both IO and ISCO.

### Intra-Subject Statistical Analysis

Table 6 shows results at the intra-subject level. The number of obtained *Improvement*, *Worsening*, and *Unchanged* for each considered parameter is reported, separately by genders, as percentages of the total AIS patients. Each parameter is also referenced to already published healthy young adults values (D’Amico et al., 2018a). Only four for males and five for females, out of 13 parameters, reach up to about 30% or just above in *Improvement*, either in the frontal or sagittal plane, i.e., absolute ASIS height difference in the frontal plane (|ΔASIS|), pelvis torsion (|PT|), sacral angle (SA), and TKA for males; averaged spinal offset (|ASO|), the primary and secondary Cobb angles (CA1, CA2), TKA, and lumbar lordosis angle (LLA) for females. However, *Worsening* shows parameters with a relevant percentage (over 30%), such as |PT| and TKA for males, |PT|, TKA, and the underfoot load difference (|ΔUL|) for females.

**TABLE 6:** Intra-subject statistical analysis comparison IO vs. ISCO posture: number of obtained for *Improvement*, *Worsening*, and *Unchanged* for each considered parameter reported, separately per genders, as percentages of the total AIS patients and healthy young adults.

		AIS patients males			AIS patients females			Healthy young adults		
Parameter	Descriptions	*Improvement*	*Worsening*	*Unchanged*	*Improvement*	*Worsening*	*Unchanged*	*Improvement*	*Worsening*	*Unchanged*
|ASO|	|Average frontal spinal offsets|	24.6%	15.8%	59.6%	33.3%	22.7%	44.0%	29.8%	20.7%	49.6%
|AGO|	|Average frontal global offsets|	24.6%	15.8%	59.6%	14.7%	16.0%	69.3%	26.4%	30.6%	43.0%
|ΔASIS|	|ΔAnterior superior iliac spine|	35.1%	17.5%	47.4%	28.0%	18.7%	53.3%	19.8%	14.0%	66.1%
|ΔPSIS|	|ΔPosterior superior iliac spine|	24.6%	10.5%	64.9%	22.7%	24.0%	53.3%	21.5%	19.0%	59.5%
CA1	1°Cobb angle	21.1%	12.3%	66.7%	33.3%	13.3%	53.3%	28.1%	23.1%	48.8%
CA2	2°Cobb angle	12.3%	22.8%	64.9%	34.7%	13.3%	52.0%	25.6%	26.4%	47.9%
|PT|	|Pelvis torsion| = |(ΔASIS-ΔPSIS)|	29.8%	29.8%	40.4%	21.3%	32.0%	46.7%	29.8%	35.5%	34.7%
SA	Sacral angle	42.1%	17.5%	40.4%	25.3%	28.0%	46.7%	35.5%	5.8%	58.7%
TKA	“Thoracic” Kyphosis angle	36.8%	36.8%	26.3%	29.3%	41.3%	29.3%	36.4%	27.3%	36.4%
LLA	“Lumbar” Lordosis angle	26.3%	24.6%	49.1%	29.3%	20.0%	50.7%	20.7%	12.4%	66.9%
|ΔUL|	|ΔUnderfoot load average|	23.3%	27.9%	48.8%	22.0%	40.7%	37.3%	22.5%	27.5%	50.0%
FPI	Frontal postural index	12.3%	3.5%	84.2%	17.3%	4.0%	78.7%	14.0%	9.9%	76.0%
SPI	Sagittal postural index	19.3%	14.0%	66.7%	14.7%	18.7%	66.7%	27.3%	10.7%	62.0%
GPI	Global postural index	7.0%	5.3%	87.7%	4.0%	2.7%	93.3%	6.6%	6.6%	86.8%

From Table 6, by simply computing the signed differences concerning the corresponding values determined in healthy young adults, it is possible to compare AIS patients’ behavior with that of healthy young adults. For example, looking at the SPI row shows that: *Improvement* percentage is 8% lower; the *Worsening* percentage is 3.3% higher, and the *Unchanged* percentage is 4.7% higher if we compare AIS males with healthy young adults’ values.

## Discussion

The paper’s overall goal was to study ISCO maneuver in AIS patients who did not receive any previous specific postural education treatment. In ISCO, a generic command (i.e., the request to assume the best correct self-perceived standing posture without adding any specific indication or feedback) was given to AIS patients, the same way it was given for healthy young adults (D’Amico et al., 2018a). The reason for this generic command was to evaluate if such patients can perceive and modify their posture and spine shape, in a spontaneous way, without previous conditioning training. Further questions were related to establishing differences by gender, if any, for AIS patients, in IO and ISCO. Finally, AIS patients’ posture was compared with healthy young adults’ posture to establish if AIS patients presented a compromised ability to perform a self-correction maneuver.

To answer the above questions, we used the advanced non-ionizing real-time optoelectronic stereophotogrammetric measuring method (D’Amico et al., 2017a) that proved to be a very accurate detailed solution in 3D posture analysis and self-correction measurement on a healthy young adult population (D’Amico et al., 2018a). The capability of such a method to properly reconstruct and measure the 3D spine shape was discussed for the first time in D’Amico et al. (1995). The agreement between the opto-electronic stereophotogrammetric spine shape reconstruction and x-ray evaluation on scoliotic patients was demonstrated in a comparative study in which both evaluations were performed within minutes of each other (D’Amico and Vallasciani, 1997). More recently, such a method was used to determine the baseline reference normative data of a healthy young adult population, i.e., the physiological standard for 30 selected quantitative 3D parameters that accurately capture and describe a full-skeleton, upright-standing attitude, including spine morphology and pelvic parameters. Such data demonstrated a high agreement with results obtained via other methods as presented in the existing literature. There is firm consistency with the results, especially concerning the spine, obtained via x-ray measurements which at this time are the “gold standard” (D’Amico et al., 2017b; Kinel et al., 2018). Other non-ionizing approaches, such as the rasterstereographic back-surface measurement technique or the recently introduced ultrasound measurement, were excluded because they raise concerns and questions that need further clarification about measurement accuracy and/or the need for the patient to keep a constrained position during the scanning measurement (Kotwicki et al., 2007; Frerich et al., 2012; Takács et al., 2014; Bassani et al., 2019; Wanke-Jellinek et al., 2019; Wu et al., 2020). To note, new recently introduced optical method and software tool (Ćuković et al., 2020) relying on the digitalized dorsal surface associated with a new 3D spine modeling (Ćuković et al., 2015), multiscale and registerable 3D generic spinal model complemented by CAD technologies show a new, hopefully promising technique to improve rastereographic clinical reliability.

Rehabilitation aims to improve the postural performance by stimulating, via proper motor exercises, the proper integration and management in all the components of the central nervous system controlling posture (Weiss et al., 2006; Czaprowski et al., 2014; Monticone et al., 2014, 2016; Kuru et al., 2016; Negrini et al., 2018, 2019; Schreiber et al., 2019). This study’s outcomes support the thesis that postural training in AIS patients is needed because of the poor self-correction ability they demonstrate, and it must be individualized. In the group analysis, by considering Table 4, it is possible to notice that females present changes in five out of thirteen parameters (38.4%) during the ISCO task. However, for the primary Cobb angle (CA1), the value of change could be considered without clinical relevance resulting in about 1°Cobb angle. Thus, by adding this latter to the unchanged parameters, the *Unchanged* percentage increases to 69.23% of the considered postural parameters.

Moreover, when parameters exhibit statistically significant and clinically relevant value changes (Table 4), they show the tendency of a postural worsening. Both genders present a substantial TKA reduction (around 9° in males and 8° in females, respectively), but such reduction induces a further departure from normative values (Table 5). Furthermore, a forward unbalancing of both the trunk and global posture is highlighted by the values of SA, averaged spinal offset sagittal (ASO SG), and averaged global offset sagittal (AGO SG) for females parameters (D’Amico et al., 2018a). Conversely, unexpectedly the lumbar level seems to be entirely neglected, in terms of perception and motor control in both genders, in that minimal changes (−0.41° for males and −0.2° for females) occur in ISCO (Table 4). Significant modifications of TKA and SA in ISCO were found in healthy young adults (D’Amico et al., 2018a) and healthy adolescents as well (Czaprowski et al., 2014). We confirmed TKA and SA relevant modifications in AIS patients, but our results did not confirm females’ better ability to modify their lumbar lordosis than males (Czaprowski et al., 2014). Indeed, we found that the lumbar spine proprioception and control are scarce in both sexes, either in AIS patients or healthy young adults. Statistically significant differences in six parameters over 13 (46.1%) showed a worse posture in AIS male patients than healthy young adults males in IO (Table 5). Conversely, AIS patients’ females were worse than healthy young adults’ females in only three parameters.

As expected, in the frontal plane, the largest and more clinically relevant discrepancies are related to spine deformities, while in the remaining parameters, AIS patients are not so different from healthy young adults, except for the underfoot load difference (|ΔUL|) in male AIS patients. Indeed, this result confirms that healthy young adults’ posture is not optimal. Asymmetry (associated with unbalanced posture, uneven underfoot loads, slight spinal curvature in the frontal plane, and pelvis torsion) appears to be a standard in healthy young subjects. Differences in the underfoot distribution between AIS patients and age and BMI matched healthy subjects have been observed during gait, showing differences between feet asymmetries of COP patterns and COP velocities related to scoliosis severity (Gao et al., 2019).

Healthy young males have the same TKA as females but a lower LLA value (D’Amico et al., 2017b, 2018a). On the contrary, LLA in scoliotic males is higher than that in healthy young adult males, and its value is the same as that of AIS females.

On the other hand, scoliotic females have reduced TKA compared with healthy young adult females, but similar LLA. During the self-correction performance, the AIS patients present a different behavior by gender. In females, disparities observed in IO compared with healthy young adults increase in number, while in males, they decrease. Indeed, AIS females worsen their posture showing an increase in the trunk’s forward leaning (ASO SG and SA) and an increased underfoot loading unbalancing (|ΔUL|). Conversely, the values of |ΔASIS| and |ΔPSIS| show that AIS males have good pelvis control. In fact, while in the IO, they presented more oblique pelvis than healthy young adults, they performed such a good self-correction that, during such maneuver, they showed no more differences in pelvis obliquity compared with healthy young adults.

Looking at summarizing indexes at intra-subject level analysis, as for healthy young adults (D’Amico et al., 2018a), a high percentage of AIS patients could not modify their 3D posture. These findings are relatively confirmed even when changes are analyzed separately in the frontal or sagittal planes. Remarkably, only 7% of males and 4% of females were able to reach a global improvement. However, when AIS patients performed self-correction maneuvers, they tended to improve those parameters related to scoliotic deformities, pelvic obliquity, and lateral leaning (i.e., where they showed worse disparities vs. healthy young adults in IO), but without reaching relevant clinical changes. Worth noting, such improvements are obtained at the cost of a clinically remarkable sagittal posture worsening: flattening in the trunk and forward posture unbalancing either in the trunk (both genders) or globally (females only).

Indeed, all the above demonstrate that, when changes occurred, participants could not focus and control their posture globally, but they could focus only on a few aspects at a time, individually. The best values of *Improvement* were obtained in the males group in SA (42.1%), TKA (36.8%), and |ΔASIS| (35.1%) while for the females in CA2 (34.7%), CA1 (33.3%), and |ASO| (33.3%). However, even *Worsening* scored high in some parameters. Curiously, males presented in TKA (36.8%) and |PT| (29.8%) the same percentages of *Improvements* and *Worsening*, respectively. Furthermore, for |ΔUL| (27.9% vs. 23.3%) and CA2 (22.8% vs. 12.3%) the *Worsening* outweighs the *Improvements*. For the females, it is possible to see in Table 6 that the *Worsening* scored higher than improvements in six out of 11 considered postural parameters with TKA and |ΔUL| *Worsening* exceeding 40%. However, the percentage of *Improvement*/*Worsening* of summarizing indexes (FPI, SPI, and GPI) resulted far below those obtained for such single postural parameters. Thus, all these results lead to the deduction that posture perception and control are not an easy task, and it is differently perceived/managed at different parts of the body among participants. The lumbar level shows the largest unmodified behavior. Based on that, it can be argued that specific, focused work, and physical activity is needed (Moon et al., 2013; Monticone et al., 2014; Berdishevsky et al., 2016; Negrini et al., 2018, 2019; Mueller and Niederer, 2020). The same kind of postural control limitation was found in healthy young adults (D’Amico et al., 2018a). For this reason, it can be argued that AIS patients do not present impaired behavior either in proprioception or motor control compared with healthy young adults.

The 3D stereo-photogrammetric approach, together with the implemented 3D entire skeleton model, allows quantifying body posture and 3D spine shape with many numerical parameters. In this study, we chose to consider 13 numerical parameters (Table 2) describing the 3D posture and spine shape of AIS patients quantifying the spinal deformities in the frontal plane and the spine curves in the sagittal plane, the global and trunk offsets in the frontal and sagittal planes, the pelvis obliquity and torsion, and the underfoot load distribution.

This approach allows describing posture focusing attention on either specific aspects or at a more global level. The 3D stereo-photogrammetric approach accuracy leads the statistical analysis to discriminate, as statistically significant, differences related to subtle changes accounted for even only about 1°Cobb angle value in spine shape, far below the level of clinical significance.

Nevertheless, there are inherent limitations in the study because we compared populations at different ages, i.e., adolescents and young adults. Results may show different outcomes if healthy adolescent would be compared, so further studies are necessary. A further limitation relates to the fact that we could not include a direct comparison between patients undergoing treatment with PSSEs techniques and a control group treated with a traditional therapy approach in our study. This will be the subject of a future study.

## Conclusion

The study’s clinical relevance is related to the finding that, as found for healthy young adults, the self-correction maneuver is not instinctive in AIS patients but must be learned with specific postural training. Participants were, in general, not able to focus and control their posture globally, but only in a few aspects at a time in an individual way. In such characteristics, AIS patients are not so different from healthy young adults. Some perception of deformity is present in AIS patients for both planes, either frontal or sagittal. There is more attention to the postural control in the sagittal plane (in that relevant modifications are observed); nevertheless, self-correction maneuver led to a worsening in this plane.

Moreover, control on the lumbar level seems to be neglected in both genders. These findings support the necessity of customized PSSEs to treat AIS patients. The personalized PSSEs should aim to stimulate the individual’s capacity to perceive and control his/her posture, and particularly the shape of the spine, to reduce spinal deformities, and the limitation of functional spinal units in order to prevent inappropriate posture and improve stability of the spine through voluntary intervention.

The 3D stereo-photogrammetric approach effectively described participants’ posture, motor control, and proprioceptive capability. Its routine usage is recommended as a complementary tool for analyzing AIS patients to design a customized PSSE therapy and monitor the treatment efficacy in producing an improved proprioceptive ability.

## Data Availability Statement

The raw data supporting the conclusions of this article will be made available by the authors, without undue reservation.

## Ethics Statement

The studies involving human participants were reviewed and approved by The Ethics Committee University of Medical Sciences, Poznan, Poland. Resolution number: 75/17. Written informed consent to participate in this study was provided by the participants’ legal guardian/next of kin. Written informed consent was obtained from the minor(s)’ legal guardian/next of kin for the publication of any potentially identifiable images or data included in this article.

## Author Contributions

All authors listed have made a substantial, direct and intellectual contribution to the work, and approved it for publication.

## Conflict of Interest

MD’A and PR own shares of the Bioengineering & Biomedicine Company Srl. This does not alter our adherence to Frontiers in Bioengineering and Biotechnology policies on sharing data and materials. The Bioengineering & Biomedicine Company Srl did not play any direct role in the study design, data collection and analysis, decision to publish, or the manuscript preparation. The remaining author declares that the research was conducted in the absence of any commercial or financial relationships that could be construed as a potential conflict of interest.

## Footnotes

1 Bioengineering & Biomedicine Company S.r.l., Pescara, Italy.

2 NaturalPoint Inc., Corvallis, OR, United States.

3 Zebris Gmbh, Isny, Germany.

## Abbreviations

AIS: adolescent idiopathic scoliosis
PSSEs: physiotherapeutic scoliosis-specific exercises
IO: indifferent orthostasis
ISCO: instinctive self-correction.
